# Effects of room temperature and cold storage on the metabolic and haemostatic properties of whole blood for acute normovolaemic haemodilution

**DOI:** 10.1371/journal.pone.0267980

**Published:** 2022-05-13

**Authors:** Junko Ichikawa, Masaki Kouta, Masako Oogushi, Makiko Komori

**Affiliations:** Department of Anaesthesiology, Tokyo Women’s Medical University Medical Centre East, Tokyo, Japan; University of Maryland Baltimore County, UNITED STATES

## Abstract

**Background:**

Acute normovolaemic haemodilution (ANH), as a blood-conservation technique, avoids the need for allogeneic blood transfusions. The historic practice of cold-storing type-O whole blood (WB) in military fields popularised the transfusion of refrigerated WB to treat acute bleeding. In this study, we compared the effects of room temperature (RT) and refrigeration up to 24 hours on the coagulation properties of WB for ANH.

**Materials and methods:**

Each WB sample, collected from 12 male volunteers, was divided into two parts, one stored at RT and the other refrigerated for 24 hours. Complete blood counts (CBC), blood gas levels, and coagulation profiles were measured, and rotational thromboelastometry (ROTEM) measurements were performed at the initial collection time point (baseline) and at 6, 12, and 24 hours after initial collection.

**Results:**

The preservation of platelet aggregation response induced by arachidonic acid and adenosine diphosphate was better in cold-stored WB compared to that in RT-stored WB. The platelet aggregation response induced by thrombin receptor-activating peptide 6 was significantly decreased in all samples after 24 hours of storage when compared with that at baseline. The lactate levels in WB stored at RT increased significantly after 6 hours of storage compared to that of cold-stored samples. There were no significant differences in CBC, coagulation parameters, and ROTEM variables between the cold-stored and RT-stored WB samples.

**Conclusion:**

WB for ANH stored in the refrigerator showed better metabolic characteristics after 6 hours of storage and better aggregation response after 12 hours of storage than WB stored at RT.

## Introduction

Acute normovolaemic haemodilution (ANH)—a blood-conservation technique—is cost-effective and reduces red blood cell loss in haemodiluted patients, thereby minimising exposure to allogeneic blood and the accompanying risk of transfusion-related infections and reactions [1, 2]. The standard storage procedures outlined by the American Association of Blood Bank recommend that whole blood (WB) for ANH can be stored at room temperature (RT) for 8 hours and refrigerated for 24 hours. The limited shelf life can be attributed to the risk of bacterial contamination and storage lesions which negatively affect *in vivo* recovery, survival, and haemostatic function. Rocking of WB is not recommended due to chances of inducing red blood cell sublethal injury and haemolysis [3]. Past studies suggested that agitation does not enhance or improve platelet function in WB [4, 5].

Although refrigerating WB for ANH is not a widespread practice, the historic military practice of cold-storing type-O WB in the battlefields expanded the use of refrigerated WB for resuscitation of patients with haemorrhagic shock [6]. Considerable haemostatic effects and minimal transfusion prerequisites of refrigerated WB are particularly beneficial in trauma resuscitation [7] and paediatric cardiac surgery [8].

However, the haemostatic properties of refrigerated WB in clinical settings do not correspond with specific assessments of functional coagulation factors and platelets in stored WB. To date, the effects of WB storage at RT and in the refrigerator for 24 hours have not been compared adequately. Few studies have assessed the effects of serial storage on various coagulation properties of refrigerated WB for longer than 24 hours [9, 10]. These studies showed that the *in vitro* integrity of plasma protein factors and platelet function do not fall below clinically useful levels in refrigerated WB, and cold-stored WB can support the short-term haemostatic needs of traumatised patients. The effects of refrigeration have been studied extensively on individual blood components such as plasma [11] and platelets [5, 12, 13]. These studies have revealed that refrigeration decreases the risk of bacterial sepsis, diminishes platelet metabolism, and may preserve haemostatic function better than standard RT storage. Therefore, we compared the effects of WB storage at RT with WB storage in the refrigerator for up to 24 hours, using metabolic analysis and coagulation and platelet function assessments to determine the optimum method of storing WB for ANH. We hypothesised that cold storing of WB for ANH—a standard practice for red blood cell storage—will maintain metabolic and functional characteristics of WB better than storage at RT.

## Materials and methods

The study was approved by the Institutional Review Board, and written informed consent was obtained from all participants. WB from 12 healthy adult male volunteers with a mean age of 28.7 years (height, 172.1 ± 5.6 cm; weight, 63.5 ± 4.5 kg) was investigated using an *in vitro* study design. All participants denied taking any medications within the previous 14 days. WB (200 mL) was drawn from the antecubital vein through an 18 G needle and was collected in standard sterile bags containing 56-mL citrate-phosphate-dextrose adenine anticoagulant (Karmi CA; Kawasumi Laboratories, Tokyo). Each WB unit was divided equally into two PVC-DEHP blood transfer bags (Karmi CA; Kawasumi Laboratories, Tokyo); one was stored for 24 hours at RT (20–24°C) and the other in the refrigerator (1–6°C) for the same period in accordance with the standard storage procedures outlined by the American Association of Blood Banks.

The time of initial WB collection was designated as the baseline. Fifteen-millilitre aliquots were taken from each bag at 6, 12, and 24 hours. Each bag was gently mixed before sampling, and the samples were obtained using 20-mL syringes via a sterile connection to maintain a closed system. For the measurement of partial pressure of oxygen (pO2) and partial pressure of carbon dioxide (pCO2), the following steps were required to avoid air exposure of the samples: removal of air bubbles, keeping sample at room temperature, and performing analysis within 10 minutes of sample collection. Complete blood count (CBC), coagulation profile, and blood gas levels were assessed, and rotational thromboelastometry (ROTEM) and ROTEM-platelet analysis were performed after the bags were left at RT for approximately 10 minutes. CBC, including platelet count, platelet distribution width, and mean platelet volume were determined, and coagulation profiles, comprising prothrombin time (PT), activated partial thromboplastin time (APTT), PT-international normalised ratio (INR), fibrinogen concentration, and antithrombin activity were analysed in the central haematological laboratory according to the institutional protocol. CBC was performed using XN-3000 (Sysmex Co, Kobe, Japan), and coagulation profiles were assessed using CS-5100 (Sysmex Co, Kobe, Japan). Blood pH and blood sodium, potassium, glucose, pCO2, pO2, bicarbonate, and lactate levels were determined using GEM Premier 4000 (Instrumentation Laboratory, Bedford, MA).

The ROTEM-platelet (TEM International, Munich, Germany) measures platelet aggregation in WB samples using a multiple impedance aggregometer with arachidonic acid (AA; final concentration, 0.42 mM) in ARATEM, adenosine diphosphate (ADP; final concentration, 10 μM) in ADPTEM, and thrombin receptor-activating peptide 6 (TRAP; final concentration, 36 μM) in TRAPTEM as agonists. The test measures the changes in impedance between two electrodes as platelets adhere and aggregate in response to an agonist. Impedance aggregometry is expressed by three parameters: amplitude at 6 minutes (in Ohm), maximum slope of aggregation curve (in Ohm/minutes), and area under the curve (AUC, in Ohm • minutes). Amplitude at 6 minutes, maximum slope of aggregation curve, and AUC reflect the extent of platelet aggregation, rate of aggregation, and overall platelet aggregation, respectively.

We tested the kinetics of formation, strength, and stability of clots formed from stored WB using the extrinsic test (EXTEM) and fibrin polymerisation analysis (FIBTEM) in ROTEM. For the EXTEM assays, the following variables were analysed: clotting time (CT [s]), clot formation time (s), alpha angle (angle of tangent at a 2-mm amplitude [°]), maximum clot firmness (MCF [mm]), and maximum lysis (%); MCF was also measured for the FIBTEM assays. The platelet component of the clot strength was assessed from the clot elasticity [14], which reflects the force that the blood clot resists during rotation within the device.

### Statistical analysis

Data were tested for normal distributions using the Shapiro-Wilk’s test. Changes in platelet aggregation, CBC, coagulation profiles, blood gas levels, and ROTEM values for each stored condition between baseline and 6, 12, and 24 hours after phlebotomy were compared using two-way analysis of variance (ANOVA) for repeated measures. Differences in platelet aggregation, CBC, coagulation profiles, blood gas levels, and ROTEM values between RT and cold storage at 6, 12, and 24 hours after initial collection were analysed by post-hoc comparisons using a paired Student’s t-test. The level of significance was adjusted according to the Bonferroni correction. The criterion for rejection of the null hypothesis was *P* < 0.05. All statistical analyses, except statistical power analyses determined using G*Power 3.1, were performed using SPSS software (version 11.0; IBM, Chicago, IL).

We conducted a power analysis based on a pilot study, that assessed a significant difference among groups according to elapsed time using repeated-measures ANOVA in response to stimulation with AA (ARATEM), ADP (ADPTEM), and TRAP (TRAPTEM). The analysis showed that 12 participants would be required in each group to achieve 80% power for a study design with four measurements between two groups. The α-level was set at 0.05, and the effect size *(f*) was set at 0.25.

## Results

**Fig 1:**
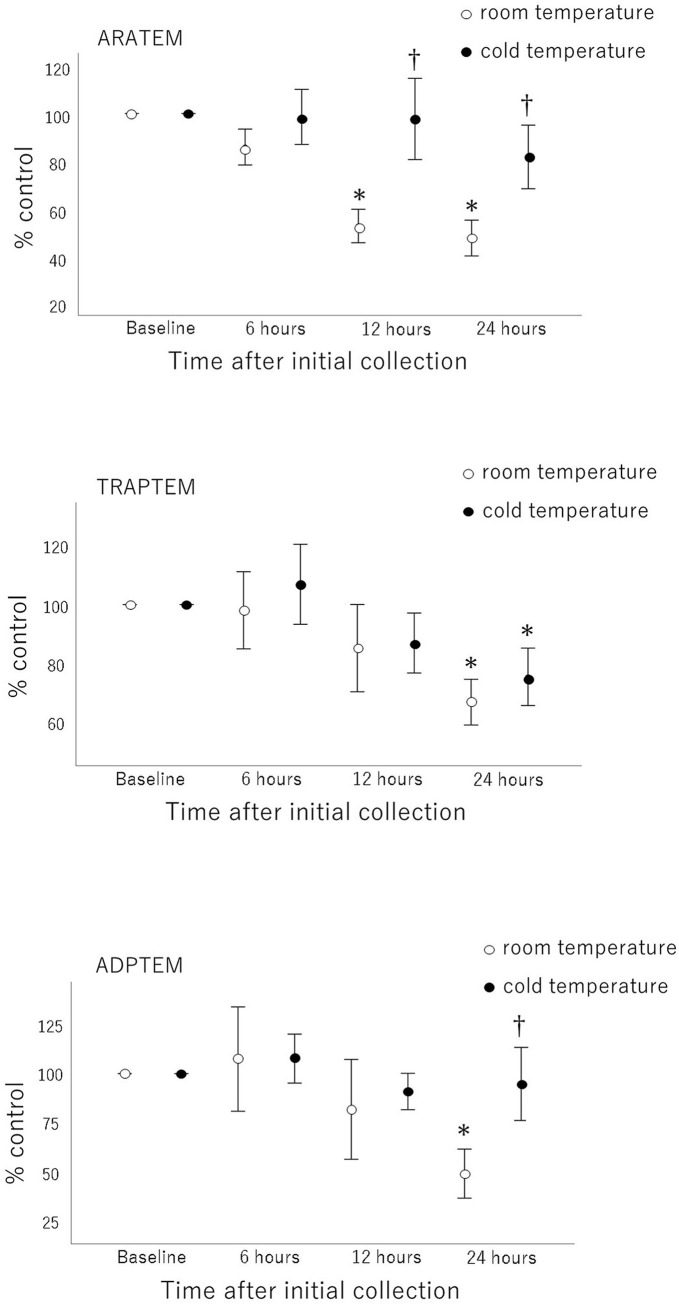
The effect of storage temperature on Area Under the Curve (AUC) induced by various agonists as measured by rotational thromboelastometry-platelet. Data are provided as the percentage of control AUC determined at baseline. Values are expressed as the mean ± standard error of the mean of independent experiments (*n* = 8) performed in duplicate determination. Differences compared to baseline (*) are shown if results from two-way analysis of variance for repeated measures are significant (*P*<0.05). ARATEM, platelet aggregation in response to stimulation with arachidonic acid; TRAPTEM, platelet aggregation in response to stimulation with thrombin receptor-activating peptide 6; ADPTEM, platelet aggregation in response to stimulation with adenosine diphosphate.

Fig 1 shows the effects of storage conditions on AUC according to the type of agonists. Data were recorded as the percentage of the control AUC determined at baseline. All chemical agonist-induced platelet aggregations were preserved up to 6 hours. AA and ADP caused no significant effects on platelet aggregation after 24 hours of cold storage, whereas there was a decrease in the response to TRAP, compared with that at baseline (P<0.05). In contrast, 24 hours after initial collection, platelet aggregations at RT induced by all chemical agonists were significantly reduced, compared with that at baseline (p<0.05). Platelet aggregation after 24 hours of cold storage was significantly higher than that after 24 hours of RT storage when stimulated with AA and ADP (P<0.05).

The effects of storage conditions on platelet aggregation parameters such as amplitude at 6 minutes, maximum slope of aggregation curve, and AUC induced by AA, TRAP, and ADP are summarised in Table 1. Data were recorded as absolute values of aggregation. Platelet aggregations after 24 hours of cold storage induced by AA and ADP were significantly higher than those after 24 hours of RT storage (P<0.05). No significant changes were observed in platelet aggregation induced by AA and ADP over the 24-hour storage period when compared with that at baseline for cold storage conditions; however, platelet aggregation induced by TRAP after 24 hours of cold storage decreased significantly when compared with that at baseline (P<0.05). Platelet aggregation induced by all chemical agonists decreased significantly after 24 hours of RT storage (P<0.05 in all cases), when compared with that at baseline.

**Table 1:** The effect of storage conditions on platelet aggregation parameters (*n* = 12).

	Baseline	Room temperature			Cold-stored(4°C)		
A6 (Ohm)	Baseline	6H	12H	24H	6H	12H	24H
ARATEM	17.2±4.2	15.2±5.8*	10.0±4.4*	9.7±4.5*	16.3±6.2	15.7±5.8^‡^	13.2±4.9**
TRAPTEM	19.8±6.0	18.2±6.0	15.1±6.1	12.8±3.7*	19.2±5.7	16.5±5.4	14.0±6.0*
ADPTEM	15.9±5.8	13.7±6.3	10.6±4.9*	6.6±2.5*	15.5±4.9	13.5±6.1	12.8±3.6**
MS (Ohm/min)							
ARATEM	5.7±1.7	4.5±1.4*	2.7±1.1*	2.5±1.4*	5.1±1.7	4.3±1.7	3.7±1.3* **
TRAPTEM	6.4±2.1	5.4±1.7	4.7±2.0	4.0±1.3*	5.5±1.6	4.5±1.3	4.1±1.4*
ADPTEM	5.3±2.6	3.8±1.9	2.6±1.3*	1.6±0.8*	4.1±1.1	3.5±1.6	3.5±1.0**
AUC (Ohm・min)							
ARATEM	66.6±16.7	59.3±20.0	36.5±15.6*	32.3±16.9*	61.5±22.9	56.5±21.4^‡^	48.2±16.1**
TRAPTEM	76.8±23.7	69.9±22.3	58.8±23.8	49.8±15.4*	71.7±21.9	60.7±19.0	52.2±20.8*
ADPTEM	55.8±17.2	50.4±23.3	35.2±16.4*	20.8±9.7*	55.1±15.9	49.5±21.6	44.0±10.8**

Data are shown as mean ± standard deviation. Differences compared to baseline (*) and time point-matched room temperature (‡,**) are shown if results from both two-way analysis of variance for repeated measures and post-hoc comparisons using a paired Student’s t-test were significant (P<0.05).

A6, amplitude at 6 minutes; MS, maximum slope of aggregation curve; AUC, area under the curve; ARATEM, platelet aggregation in response to stimulation with arachidonic acid; TRAPTEM, platelet aggregation in response to stimulation with thrombin receptor-activating peptide 6; ADPTEM, platelet aggregation in response to stimulation with adenosine diphosphate; 6H, 6 hours; 12H, 12 hours; 24H, 24 hours

**Fig 2:**
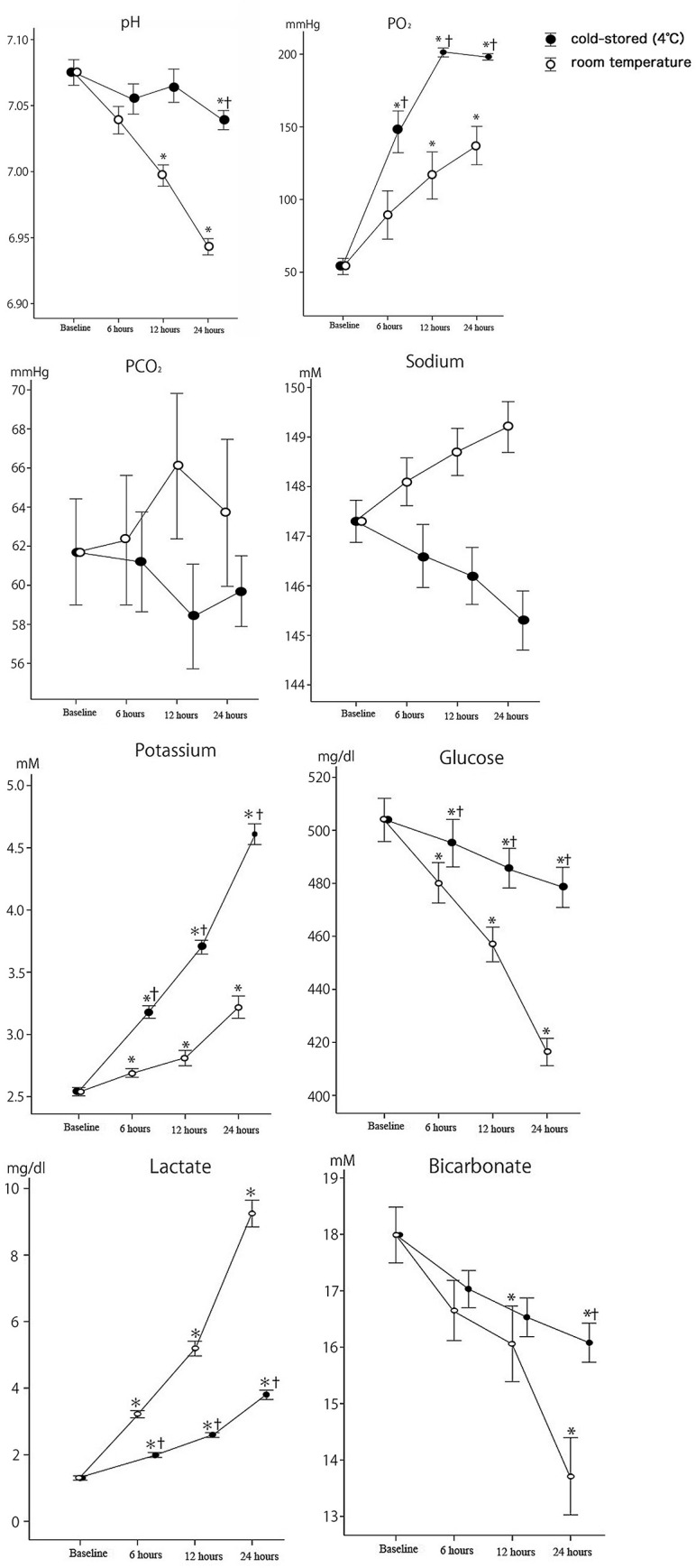
The mean values of blood gas analysis during storage (*n* = 10). Data are represented as mean ± standard error of the mean. Differences compared to baseline (*) and time point-matched room temperature (^†^) are shown if results from both two-way analysis of variance for repeated measures and post-hoc comparisons using a paired Student’s t-test are significant (*P*<0.05).

Fig 2 presents the mean blood gas levels during storage. All samples maintained acceptable levels of pH > 6.2, which is the current limit set for platelet component by the Japanese Red Cross Society. We observed an increase in lactate and a corresponding decrease in bicarbonate levels in all samples. The lactate levels in WB stored at RT increased by 229%, 371%, and 664% (P<0.05 in all cases), and bicarbonate levels decreased by 5%, 9% (P<0.05), and 22% (P<0.05) after 6, 12, and 24 hours, respectively. In comparison, the lactate levels in cold-stored WB increased by 135%, 186%, and 271% after 6, 12, and 24 hours, respectively (P<0.05 in all cases), and the bicarbonate levels did not change significantly with a decrease of 9% after 24 hours. The glucose consumption of RT-stored WB was significantly higher compared to that of cold-stored WB (P<0.05), but the yielding carbon dioxide rates were comparable for the RT and cold-stored samples. The dissolved oxygen levels were significantly elevated, especially in cold-stored samples (P<0.05), suggesting adequate gas exchange during storage. Limited but significant increase in potassium levels was observed in all samples at 12 and 24 hours after phlebotomy (P<0.05), and the potassium levels in cold-stored samples were significantly higher than those in RT samples (P<0.05).

Table 2 presents the mean CBC and coagulation profiles during both types of storage. There were no significant differences in haematocrit, haemoglobin levels, red blood cell counts, platelet counts, platelet distribution width, mean platelet volume, APTT, PT, PT- INR, fibrinogen levels, and antithrombin activity between the WB samples under each storage condition. Although there were significant increases in APTT, PT, and PT-INR in both groups as time progressed, all sample values were within the reference range.

**Table 2:** The mean complete blood counts and coagulation profiles during storage (*n* = 9).

		Room temperature			Cold-stored(4°C)		
	Baseline	6H	12H	24H	6H	12H	24H
Red blood cell count (10^4^/μl)	428.4±22.5	427.2±20.8	427.0±20.1	427.4±18.9	433.1±25.9	431.0±22.8	434.2±23.5
Hemoglobin (g/dl)	13.6±0.7	13.5±0.8	13.6±0.8	13.6±0.8	13.8±0.9	13.6±0.7	13.8±0.8
Hematocrit (%)	40.5±2.9	40.1±1.5	41.1±2.1	41.7±2.0	40.3±2.3	40.3±2.2	40.4±2.1
Platelet count (10^10^/l)(15.8–34.8)	23.2±4.3	22.4±4.6	24.5±4.8	22.9±4.5	22.4±4.5	22.9±4.6	21.4±4.2
PDW (fL)	12.0±1.9	10.8±1.1	12.7±1.4	11.1±1.4	12.0±1.5	12.4±1.5	11.9±1.5
MPV (fL)(9.2–12)	10.3±0.8	9.8±0.5	10.5±0.6	10.2±0.5	10.2±0.7	10.5±0.7	10.3±0.6
PT(s)	11.5±0.4	11.8±0.5*	12.0±0.5*	12.1±0.5*	11.6±0.3*	11.8±0.4*	11.8±0.4*
PT/INR	0.99±0.05	1.01±0.05*	1.04±0.05*	1.05±0.05*	1.00±0.03*	1.02±0.03*	1.02±0.04*
APTT (s)	31.0±2.8	31.8±3.7	33.2±4.0*	33.9±4.2*	31.3±3.0*	32.4±3.5*	33.0±3.2*
Fibrinogen (mg/dl)	244.1±31.8	242.4±38.1	244.2±36.6	246.2±37.2	246.1±33.2	247.1±35.4	246.3±34.1
Antithrombin activity (U/ml)	104.4±11.9	103.0±12.3	102.4±11.3	102.9±11.4	107.1±12.5	106.6±12.3	106.9±11.8

Data are shown as the mean ± standard deviation. Differences compared to baseline (*) are shown if results from two-way analysis of variance for repeated measures are significant (P<0.05).

PDW, platelet distribution width; MPV, mean platelet volume; PT, prothrombin time; INR, international normalised ratio; APTT, activated partial thromboplastin time; 6H, 6 hours; 12H, 12 hours; 24H, 24 hours

The WB coagulation parameters assessed by ROTEM are presented in Table 3. All ROTEM variables were within the normal range for adults during both storage conditions. However, CTs higher than the standard values observed at baseline were evident in some units. Both storage conditions caused no significant changes in the ROTEM variables observed at baseline and after the 24-hour storage period. There were no significant differences between the RT and cold-stored samples in terms of any of the ROTEM parameters. Maximum lysis values in all units were maintained at <15%, indicating that fibrinolysis was not induced during storage.

**Table 3:** Results of whole blood coagulation measured by rotational thromboelastometry during storage (n = 10).

	Normal range		Room temperature			Cold-stored(4°C)		
EXTEM		Baseline	6H	12H	24H	6H	12H	24H
CT(s)	38–79	83.8±13.2	77.8±9.8	80.8±8.0	85.2±10.3	80.7±14.1	75.4±8.2	82.9±8.1
CFT(s)	34–159	105.3±20.1	105.9±17.4	117.0±22.1	105.5±12.2	108.3±13.4	109.6±17.0	114.6±14.1
α (°)	63–83	70.0±3.6	68.9±4.1	67.8±3.9	68.0±4.1	68.4±2.5	68.3±3.2	68.7±3.5
MCF (㎜)	50–72	60.9±4.2	59.8±2.8	59.3±4.0	58.7±3.3	59.5±3.3	59.5±4.2	58.5±3.7
MCE (dimensionless)	100–257	158.0±25.5	149.8±16.6	147.8±23.5	143.6±19.9	148.3±18.9	149.3±25.6	142.7±22.0
ML (%)	0–15	0	1(4.3)	0(1.5)	1 (3.0)	1.0 (4.25)	2.0 (4.5)	3.0 (3.5)
FIBTEM								
MCF (mm)	9–25	12.3±2.0	11.5±1.9	11.3±2.5	10.7±2.1	11.2±4.2	10.8±3.1	10.3±2.4
MCE (dimensionless)	10–33	14.1±2.6	13.0±2.4	12.8±3.2	12.0±2.6	12.9±5.9	12.2±4.2	11.6±3.0
MCEplatelet	66–247	143.9±25.9	136.7±15.8	135.0±22.8	131.5±18.0	135.4±18.7	137.1±24.8	131.2±20.4

Data are shown as the mean ± standard deviation or median (Interquartile range).

EXTEM, extrinsic test; FIBTEM, fibrin polymerisation assays; CT, clotting time; CFT, clot formation time; α, angle of tangent at a 2-mm amplitude; MCF, maximum clot firmness; MCE, maximum clot elasticity; ML, maximum lysis; 6H, 6 hours; 12H, 12 hours; 24H, 24 hours

## Discussion

In this study, citrate-phosphate-dextrose adenine anticoagulated WB refrigerated for 24 hours showed better AA and ADP-induced platelet aggregation and metabolism than WB stored at RT. The ROTEM results, platelet counts, and fibrinogen levels were stable for cold-stored WB and were comparable to the changes observed in WB stored at RT.

The markedly reduced ability of platelets stored at RT to aggregate upon stimulation with AA and ADP may be attributed to the progressive deterioration of the energy generating mechanism [15]. The transition of platelets from a quiescent to activated state increases adenosine triphosphate demand.

In contrast, platelets stored at 4°C exhibit better aggregation when stimulated by AA and ADP than those stored at RT after 12 hours of storage, which is consistent with previous reports [9, 10], and is possibly caused by cold-induced platelet activation triggered by a rise in intracellular calcium. Calcium influx activates specific signalling pathways and facilitates release of granule contents, amplifying platelet activation during cold storage [16]. Active glycoprotein IIb/IIIa receptors play a central role in the cross-linking of fibrinogen or Von Willebrand factor among receptors to mediate platelet aggregation [17]. A similar decline in aggregation induced by TRAP at both storage temperatures may be caused by a reduction in the number of high-affinity thrombin-binding sites, inducing platelet secretion and aggregation [18].

Glucose consumption was slower for cold-stored WB than that for the RT counterpart, which concurs with the lower glycolytic metabolism of cold-stored WB [9]. Lactate levels increased gradually in the cold-stored WB, whereas significantly elevated lactate levels were observed in the RT-stored WB after 6 hours. The glycolytic pathway is accelerated during ex-vivo storage of WB under hypoxic conditions, leading to consumption of glucose and consequent accumulation of lactate and free hydrogen ions, which are buffered by bicarbonate to yield carbon dioxide and water [19].

The metabolic assay data of this study showed signs of storage lesions at as early as 6 hours of storage at RT, which may have caused the changes in platelet aggregation. When the supply and demand of energy are imbalanced, platelets rapidly lose their capacity to respond to aggregation- and secretion-inducing agents [20]. Stable pH and glucose levels preserve platelet viability against activating stimuli caused by storage [21, 22]. Thus, the nearly constant pH and glucose levels of platelets in cold-stored WB indicated that the quality of platelets maintained by refrigeration is better.

WB aggregometry is particularly dependent on platelet count even within the normal range [23]. Although platelet counts in cold-stored and RT-stored WB are nearly similar, platelets stored at RT lose their ability to aggregate to chemical stimuli. Thus, the activation state attained by circulating platelets may differ from that attained during storage, which is related to aging and time-dependent structural and functional changes indicative of platelet storage lesions.

Mean platelet volume and platelet distribution width remained unchanged in both RT and cold-stored samples, suggesting no significant changes in the size of single platelets and platelet size distribution, a finding in contrast with those of previous studies [12, 24], where refrigeration induced changes in platelet shape from disks to spheres in 24 hours. However, these studies assessed platelet concentrates (PC) prepared from WB or buffy coats. Thus, the difference between WB assays and diluted PCs must be considered, because WB has significant pH-buffering capacity [25], which affects platelet morphology and in-vivo recovery.

Our data showed that PT, APTT, fibrinogen level, and antithrombin activity in WB stored at both temperatures for ANH were maintained within normal reference limits over the 24-hour storage period. This observation is consistent with that of a previous study [11] where most coagulation proteins stored in WB were not significantly depleted and were maintained above the lower reference limit for at least 24 hours, which is the permissible storage time before component preparation in some countries, including the United Kingdom and Canada.

In our study, the average EXTEM CTs of RT and cold-stored WB samples over time were slightly higher than the reference range; however, the differences would not be clinically significant as the ROTEM-guided transfusion algorithm recommends the administration of coagulation factors when the EXTEM CT exceeds 100 s. The platelet component of clot strength, measured by clot elasticity in both stored samples remained constant, showing that the clotting ability of cold-stored platelets was comparable to that of platelets stored at RT, despite better metabolic indices and aggregation response. The discrepancy between progressively reduced platelet aggregation response and constant platelet components as a result of ROTEM could be attributed to the sensitivity of viscoelastic tests to platelet function. Clot firmness is directly proportional to platelet counts and is associated with platelet function depending on the transmission of platelet contractile force to fibrin [26], which is the final step in platelet aggregation, mostly mediated by glycoprotein IIb/IIIa receptors. However, the thrombin formed extensively by the activators of viscoelastic tests interacts with protease-activated receptors and bypasses other pathways. Therefore, ROTEM is not sensitive to drugs acting on the thromboxane pathway or the P2Y12 ADP-dependent receptors and does not reflect impaired platelet function [27].

This study had some limitations. First, it was based solely on in-vitro experiments conducted under static conditions, which could result in different platelet functions and kinetics when administered in vivo. Thus, our observations should be complemented with clinical trials to compare the in-vivo properties of RT and cold-stored WB for haemostasis during surgery. Second, cold storage induces many changes in platelets not corresponding with aging or storage lesions of platelets stored at RT [28–30]. These changes can be influenced by several factors such as the levels of surface-receptor modifications, platelet-activation markers, and thrombotic microparticles, that were not measured herein. Third, we did not carry out any bacterial analyses after 24 hours, although this timeframe would not have provided enough time for proliferation. Considering platelet enhancement of bacterial growth during RT storage, cold storage may be more effective in reducing the risk of transfusion-related sepsis [31]. Finally, in present study, metabolic characteristics and platelet aggregation responses after storage in WB were specifically analyzed in male Japanese volunteers. Growing evidence suggest sex differences affect platelet function and coagulation factor activity [32] and genetic polymorphisms in blood donor potentially impact storage lesions [33]. Consequently, our findings may not be generalizable to women and/ or people of other racial/ethnic backgrounds.

This study demonstrated that RT-stored WB showed the development of storage lesions after 6 hours and lost platelet aggregation response after 12 hours of initial collection compared with cold-stored WB, as determined by blood gas levels, coagulation profiles, and aggregation responses. These data suggest that cold-stored WB has better metabolic and functional competence than RT-stored WB, although a more comprehensive analysis of coagulation profiles and haemostatic effects in clinical settings is required to confirm our findings.

## Supporting information

S1 Data (XLS)

S2 Data (XLS)

S3 Data (XLS)

S4 Data (XLSX)
